# Multi-Cancer Genome Profiling for Neurotrophic Tropomyosin Receptor Kinase (NTRK) Fusion Genes: Analysis of Profiling Database of 88,688 Tumors

**DOI:** 10.3390/cancers17132250

**Published:** 2025-07-04

**Authors:** Hinano Nishikubo, Kyoka Kawabata, Saki Kanei, Rika Aoyama, Dongheng Ma, Tomoya Sano, Daiki Imanishi, Takashi Sakuma, Koji Maruo, Canfeng Fan, Yurie Yamamoto, Masakazu Yashiro

**Affiliations:** 1 Molecular Oncology and Therapeutics, 1-4-3 Asahimachi, Abeno-ku, Osaka 545-8585, Japan; sn23089k@st.omu.ac.jp (H.N.); sn23131y@st.omu.ac.jp (K.K.); sn23107b@st.omu.ac.jp (S.K.); si22406x@st.omu.ac.jp (R.A.); sb24524y@st.omu.ac.jp (T.S.); sy23003h@st.omu.ac.jp (D.I.); so22500y@st.omu.ac.jp (T.S.); r21764m@omu.ac.jp (K.M.); sy23126n@st.omu.ac.jp (C.F.); m9563702@med.osaka-cu.ac.jp (Y.Y.); 2 Cancer Center for Translational Research, Osaka Metropolitan University, Graduate School of Medicine, 1-4-3 Asahimachi, Abeno-ku, Osaka 545-8585, Japan

**Keywords:** *NTRK* gene, fusion, multi-cancer genome profiling, solid tumors, RNA sequencing

## Abstract

**Background/Objectives**: The neurotrophic tropomyosin receptor kinase (NTRK) genes *NTRK1*, *NTRK2*, and *NTRK3* encode tyrosine kinase receptors, and their fusion genes are known as the oncogenic driver genes for cancer. This study aimed to compare the diagnostic ability of *NTRK* fusion among five types of multi-cancer genome profiling tests (multi-CGP tests) and determine a useful multi-CGP test for *NTRK* fusion, recorded in the Center for Cancer Genomics and Advanced Therapeutics (C-CAT) database in Japan. This study aimed to compare the diagnostic results for NTRK fusions among the five different CGP tests. **Methods**: A total of 88,688 tumor cases were enrolled in the C-CAT profiling database from 2019 to 2024. The detection frequency of *NTRK* fusion genes was compared to the results for five multi-CGP tests: NCC Oncopanel, FoundationOne CDx (F1), FoundationOne Liquid (F1L), GenMineTOP (GMT), and Guardant360. **Results**: *NTRK* fusion genes were detected in 175 (0.20%) of the 88,688 total cases. GMT, which is equipped with RNA sequencing function, frequently detected *NTRK* fusion genes (20 of 2926 cases; 0.68%) in comparison with the other four multi-CGP tests that do not have RNA sequencing analysis. GMT showed significantly (*p* < 0.05) higher diagnostic ability for *NTRK* fusions compared with the other four multi-CGP tests. Especially, *NTRK2* fusion was significantly (*p* < 0.001) more highly determined by GMT than it was by the other four multi-CGP tests. The detection rates for *FGFR1* and *FGFR3* were significantly higher in GMT than in other multi-CGP tests. In contrast, the detection rates of the *ALK* and *RET* fusion genes were significantly higher in F1L. **Conclusions**: GMT, which is equipped with RNA sequencing analysis, might show a useful diagnostic ability for *NTRK* fusions, especially for *NTRK2* fusion genes.

## Simple Summary

Neurotrophic tropomyosin receptor kinase (*NTRK*) genes are known as oncogenic driver genes for various types of cancers. Because the *NTRK* fusion rate has been reported to be only around 0.2% of all solid tumors, it is necessary to determine useful multi-cancer genome profiling tests for *NTRK* fusion. This study aimed to determine a useful multi-CGP test for *NTRK* fusion, recorded in the Center for Cancer Genomics and Advanced Therapeutics (C-CAT) database in Japan. A total of 88,688 tumor cases were enrolled in the C-CAT profiling database from 2019 to 2024. The detection frequency of *NTRK* fusion genes was compared to the results for five multi-CGP tests. *NTRK* fusions were detected in 175 (0.20%) of all cases. GenMineTOP frequently detected *NTRK* fusion genes (20 of 2926 cases; 0.68%). GenMineTOP, which is equipped with an RNA sequencing function, might show a useful diagnostic ability for *NTRK* fusions, especially for *NTRK2* fusion genes.

## 1. Introduction

*Neurotrophic receptor tyrosine kinase* (*NTRK*) genes code for tropomyosin receptor kinases (Trk) that belong to the neurotrophic factor receptor family. *NTRK1*, *NTRK2*, and *NTRK3* encode three transmembrane protein receptors called TrkA, TrkB, and TrkC, respectively. Trks are proteins involved in the differentiation and maintenance of nerve cells. They are localized in the central nervous system (CNS), peripheral nervous system (PNS), and smooth muscle and play an important role in the development and survival of sensory neurons. When any of the *NTRK1*, *NTRK2*, or *NTRK3* genes fuses with other genes, Trk proteins are overproduced. These proteins bind to ATP to promote the development and proliferation of cancer [1,2,3]. *NTRK* gene fusions have been found to be genetic abnormalities frequently detected in infantile fibrosarcoma and salivary gland secretory carcinoma. But they are extremely rare in general malignant tumors, occurring in fewer than 1% of cases [4,5,6]. Currently, there are two types of Trk/NTRK inhibitors, Entrectinib and Larotrectinib, approved in Japan. These inhibitors are permitted for use only in *NTRK*-fusion-positive solid tumors [7]. Trk/NTRK inhibitors suppress cell proliferation by specifically blocking the ATP-binding site of Trk fusion proteins. In particular, Larotrectinib has been approved as a Trk/NTRK inhibitor for both adults and children [8]. Both inhibitors have been shown to have useful inhibitory activity in patients with *NTRK*-fusion-positive disease. They have also been shown to induce high response rates, regardless of tumor organ, histology, or *NTRK* fusion partner. Although *NTRK* gene fusions are rare (<1%), highly effective drugs are available [9]. *NTRK* gene fusions can be detected by a variety of methods, such as the detection of TrkA, TrkB, and TrkC proteins by immunohistochemical (IHC) staining; the detection of gene fusions by DNA fluorescence in situ hybridization (FISH); the detection of fusions only for known translocation partners and known breakpoints by reverse transcription polymerase chain reaction (RT-PCR); and the detection of fusion genes by next-generation sequencing (NGS) [10,11]. Among these detection methods, the detection of *NTRK* fusion genes is essential for the use of Trk/NTRK inhibitors in Japan and is often performed through multi-comprehensive cancer genomic profiling (multi-CGP) tests [12].

Recently, precision medicine, which analyzes tumor genes and provides advanced treatments based on abnormalities, has become mainstream. In Japan, multi-CGP tests are being clinically applied under insurance for patients with solid tumors and locally advanced or metastatic solid tumors for which there are no standard treatments. The genomic and clinical information of patients who have undergone multi-CGP tests in Japan is collected and managed in a database called the Center for Cancer Genomics and Advanced Therapeutics (C-CAT). As of 31 December 2024, 88,688 patients are enrolled in C-CAT [13,14]. Only facilities authorized to use C-CAT can access this information through the data sharing system of the C-CAT Research Use Portal (https://www.ncc.go.jp/en/c_cat/use/index.html (accessed on 22 May 2025)).

Currently, there are five types of multi-CGP tests covered by insurance in Japan: FoundationOne CDx (F1), FoundationOne Liquid CDx (F1L), GenMineTOP (GMT), NCC Oncopanel (NCC), and Guardant360 (G360). Each multi-CGP test detects *NTRK* gene fusions in different regions. NCC and GMT can detect all regions of *NTRK1/2/3*, but F1/F1L can detect all exon regions of *NTRK1*, and G360 can detect only NTRK1. Thus, the detection ability of each test for NTRK abnormalities is different. Because the detection rate of *NTRK* gene fusions is low, it is necessary to determine which multi-CGP tests are useful among those with different detection capabilities. Therefore, this study aimed to compare the detection frequency of *NTRK* gene fusion among five types of multi-CGP tests using 88,688 solid tumor cases registered in the C-CAT database and to determine a useful multi-CGP test.

## 2. Materials and Methods

### 2.1. Multi-CGP Tests and C-CAT

The genetic alteration of a tumor was examined by one of five MGPTs: FoundationOne^®^ CDx (F1; Foundation Medicine Inc., Cambridge, MA, USA), FoundationOne^®^ Liquid CDx (F1L; Foundation Medicine Inc.), GenMineTOP^®^ Cancer Genome Profiling Systems (GMT; KONICA MINOLTA REALM Co., Inc., Tokyo, Japan), OncoGuide™ NCC Oncopanel System (NCC; Sysmex Co., Ltd., Kobe, Japan), or Guardant360^®^ CDx sequencing technology (G360; Guardant Health, Palo Alto, CA, USA). Each patient was examined using one of these 5 CGP tests. NCC, F1, and GMT were used to analyze the nucleic acids of the cancer tissue, and F1L and G360 analyzed the circulating tumor DNA in the blood. The NCC examination was tested for 114 genes variants [15]; the F1 examination was used for 324 gene variants [16]; the F1L examination, which extracts circulating tumor DNA from the blood, was used for 324 gene variants [17]; the GMT examination was used in testing for 737 gene variants [18].

**Figure 1:**
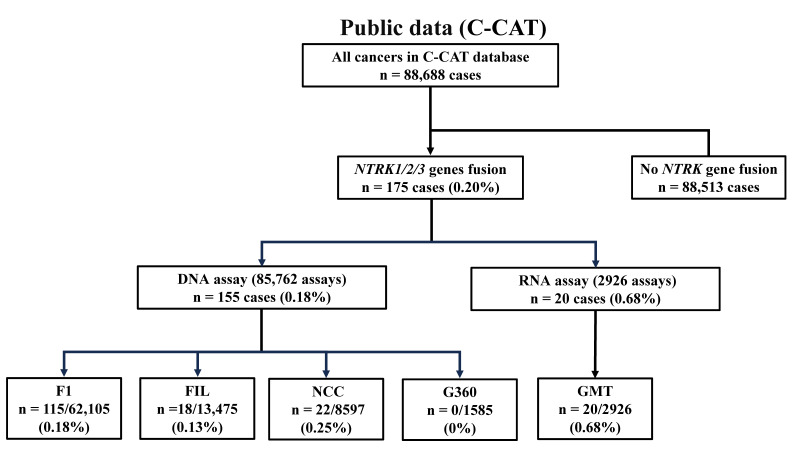
Overview of cases. Of the 88,688 cases registered in C-CAT, *NTRK* fusion genes were detected in 175 cases. Of the 175 cases, 155 cases were determined by DNA assay and 20 cases were determined by RNA assay. The detection rates for each multi-CGP test by DNA assay were 0.18% (115 of 63,105 cases) for F1, 0.13% (18 of 13,475 cases) for F1L, 0.25% (22 of 8597 cases) for NCC, and 0% (0 of 1517 cases) for G360. The detection rate by RNA assay was 0.68% (20/2926 cases) for GMT.

This study used a total of 88,688 cases registered in the C-CAT database from June 2019 to December 2024. The data analyzed included 62,105 cases of F1, 13,475 cases of F1L, 2926 cases of GMT, 8597 cases of NCC, and 1585 cases of G360 (Figure 1). This study was approved by the C-CAT review committee (C-CAT management number: CDU2022-044N) and by the medical ethics committee of Osaka Metropolitan University (approval number 2022-111).

### 2.2. Extraction of Genetic Abnormalities

Multi-CGP tests use next-generation sequencers (NGSs) to detect nucleotide substitutions, insertion/deletion mutations, gene amplifications/deletions, and gene fusions in cancer-related genes. Among the genes extracted from cancer gene panel tests, only those evaluated as pathogenic, likely pathogenic, oncogenic, or likely oncogenic in the clinical annotation of C-CAT findings were extracted. In this study, genes were selected to evaluate the true targets, and variants of uncertain significance (VUS) were not included. Pathogenic, likely pathogenic, oncogenic, likely oncogenic, and a variant of uncertain significance (VUS) were evaluated based on the definitions outlined in the interpretation guidelines for sequence variants established by a joint consensus recommendation from the American College of Medical Genetics and Genomics and the Association for Molecular Pathology. Oncogenic/likely oncogenic classifications follow C-CAT’s proprietary clinical significance criteria. These classifications serve as evidence demonstrating the associations between genetic abnormalities and cancer, based on database analysis and research findings.

### 2.3. Statistical Analysis

Significance tests were conducted using the chi-square test or Fisher’s exact probability test. A *p*-value of <0.05 was defined as statistically significant in all tests. Statistical analyses were performed using SPSS^®^ version 28 (IBM Corp., Armonk, NY, USA) and the EZR (Easy R) software package version 1.65 (Saitama Medical Center, Jichi Medical University, Saitama, Japan).

## 3. Results

### 3.1. Patient Background of Cases with NTRK Fusion Genes

Gene fusions in the *NTRK1*, *NTRK2*, or *NTRK3* genes were detected in 175 cases (0.20%) of the 88,688 cases by multi-CGP (Figure 1). *NTRK1* fusions were detected in 83 cases (0.09%), *NTRK2* fusions in 21 cases (0.02%), and *NTRK3* fusions in 71 cases (0.08%). The ages of the 175 cases analyzed ranged from 0 to 88 years, and 16.9% (*n* = 29) of these cases were younger than 20 years (Supplementary Materials, Table S1). This represents 1.74% (29 of 1670 cases) of cases under 20 years of age tested by multi-CGP, which is more frequent than previously reported. On the other hand, the detection rate of *NTRK* fusion genes in cases aged 20 years or older was 0.17% (146 of 87,018 cases), which was significantly lower than that in cases younger than 20 years of age (*p* < 0.001). When NTRK fusion genes were detected, the tissue had been submitted for multi-CGP tests in most cases (89.7%; 157 of 175 cases). The submitted tissues were collected by surgery or biopsy, and *NTRK* fusion genes were detected not only from the primary tumor but also from metastatic tumors. The average time between specimen collection and the date of request for multi-CGP tests was 388 days (range 0–3040 days), and most detections were made in tissues assessed less than one year after collection. Detection of *NTRK* fusion genes was not limited to tissues but was also conducted in 18 cases of blood that were submitted (Table 1).

### 3.2. Detection Rate of NTRK Fusion Genes in Each Multi-CGP Test

**Figure 2:**
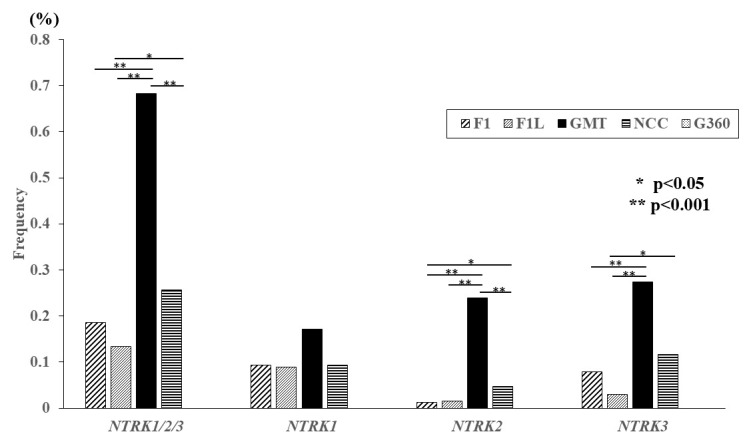
The detection frequency of *NTRK* gene fusions for each multi-CGP test. When comparing *NTRK* gene fusions overall, GMT had the highest detection rate, and there was a significant difference between GMT and F1, F1L, and NCC (*p* < 0.001). In addition, between F1L and NCC, the detection rate was significantly higher in NCC than in F1L (*p* < 0.05). When comparing the detection rates for each *NTRK* gene, GMT had the highest detection rate, although there was no significant difference in *NTRK1*. It was also revealed that GMT had the highest detection rate for *NTRK2* and had a significantly higher detection rate than F1, F1L, and NCC. *NTRK3* was also frequently detected by GMT, and there was a significant difference between GMT and F1 and F1L (*p* < 0.001). There was also a significant difference between F1L and NCC (*p* < 0.05). * *p* < 0.05; ** *p* < 0.001.

In the analysis of cases in which *NTRK1/2/3* fusion genes were detected, the detection rate was 0.18% (155 of 85,762 cases) for DNA testing and 0.68% (20 of 2926 cases) for RNA testing (Figure 1), indicating that RNA testing had a higher detection rate. The detection rate of each multi-CGP test was highest for GMT (0.68%; 20 of 2926 cases), and significant differences were observed among the three multi-CGP tests except for G360 (*p* < 0.001). There was also a significant difference in detection rate between F1L (0.13%; 18 of 13,475 cases) and NCC (0.25%; 22 of 8597 cases), with NCC demonstrating a higher positivity rate (*p* = 0.03). The detection rate of *NTRK1* was highest for GMT (0.17%; 5 of 2926 cases), while little difference was found in the detection rates for F1 (0.09%; 58 of 62,105 cases), F1L (0.09%; 12 of 13,475 cases), and NCC (0.09%; 10 of 8597 cases). Although there was no significant difference in the detection rate of *NTRK1* between the multi-CGP tests, the detection rate of GMT was nearly twice as frequent as those of the other four multi-CGP tests. The detection rate of *NTRK2* was highest in GMT (0.24%; 7 of 2926 cases), followed by NCC (0.05%; 4 of 8597 cases), F1 (0.01%; 8 of 62,105 cases), and F1L (0.01%; 2 of 62,105 cases). GMT had a significantly higher detection rate than the other three multi-CGP tests (*p* < 0.001). There was also a significant difference between F1 and NCC, with NCC being more frequently detected (*p* = 0.02). The detection rates of *NTRK3* were 0.08% (49 of 62,105 cases) in F1, 0.03% (4 of 13,475 cases) in F1L, 0.27% (8 of 2926 cases) in GMT, and 0.12% (10 of 8597 cases) in NCC. The detection rate was significantly higher in GMT than in F1 and F1L (*p* < 0.001). Although there was no significant difference in the detection rate between GMT and NCC (*p* = 0.06), it was detected more frequently in GMT (Figure 2).

### 3.3. Cases That Reached Treatment After Multi-CGP Tests

Of the 175 cases registered in the C-CAT data, 90 cases were examined by EP and administered Trk/*NTRK* inhibitors. Of these, 41 cases (49.3%), 12 cases (57.1%), and 37 cases (52.1%) were administered drugs against *NTRK1*, *NTRK2*, and *NTRK3*, respectively. Of these, 25 cases (61.0%) of *NTRK1*, 5 cases (41.7%) of *NTRK2*, and 17 cases (45.9%) of *NTRK3* achieved disease control (defined as achieving CR, PR, or SD). There was no significant difference in response rate between *NTRK* in these cases. Of the 47 cases that achieved disease control, 44 were tissue samples submitted for multi-CGP tests, and 28 cases were collected from the primary tumor and 16 cases from metastatic lesions. In addition, three cases (16.6%) of blood samples achieved disease control, including complete response, partial response, or stable disease.

### 3.4. Organs with NTRK Fusion Genes and Their Fusion Partners

**Figure 3:**
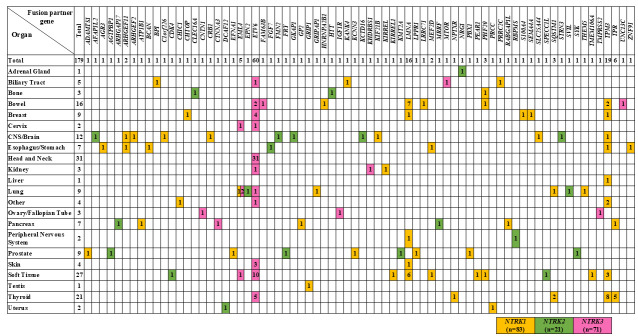
Organs with detected *NTRK* fusion genes and fusion partners. The organs and fusion partners of 175 cases in which *NTRK* fusion genes were detected are shown. Among patients with *NTRK1* fusion genes, three cases were identified in which the *NTRK1* gene was fused to two different genes within the same patient. Furthermore, among patients with *NTRK3* fusion genes, one case was identified in which the *NTRK3* gene was fused to two different genes within the same patient. The organ in which *NTRK1* gene fusions were most frequently detected was the thyroid (*n* = 16), followed by soft tissue (*n* = 14) and intestine (*n* = 12). The fusion partner was *TPM3* (*n* = 19, 22.9%), with eight cases in the thyroid. The next most common fusion partner gene was *LMNA*, with seven cases in the intestine and six cases in the soft tissue. *NTRK2* gene fusions were detected in the PNS/brain and prostate, but no common partner genes were found. *NTRK3* gene fusions were frequently detected in the head and neck and soft tissues, with the majority of fusion partner genes being *ETV6* in both organs.

*NTRK1/2/3* gene fusions were identified in 22 kinds of solid tumors, with the highest frequency in the head and neck (31 of 2866 cases; 1.08%), followed by soft tissue (27 of 3461 cases; 0.78%), thyroid (20 of 887 cases; 2.25%), intestine (16 of 14,611 cases; 0.11%), and CNS/brain (10 of 2687 cases; 0.37%). All fusions detected in the head and neck, where *NTRK3* gene fusions were most frequently identified, were *ETV6-NTRK3*. Mammary analog secretory carcinoma of salivary gland origin was the most common histological type of head and neck tumor, accounting for approximately half of cases (15 of 31 cases). Next, in soft tissues, where fusions were frequently detected, fusions were observed in *NTRK1*/*2*/*3*. *LMNA* was the most common partner gene for *NTRK1*, and *ETV6* was the most common partner gene for *NTRK3*. Focusing on *NTRK2*, it was frequently detected in the CNS/brain and prostate, but no common fusion partners were found, and the partner genes were diverse (Figure 3).

### 3.5. Detection Frequency of Other Fusion Genes, ALK, RET, ROS1, and FGFR1/2/3

**Figure 4:**
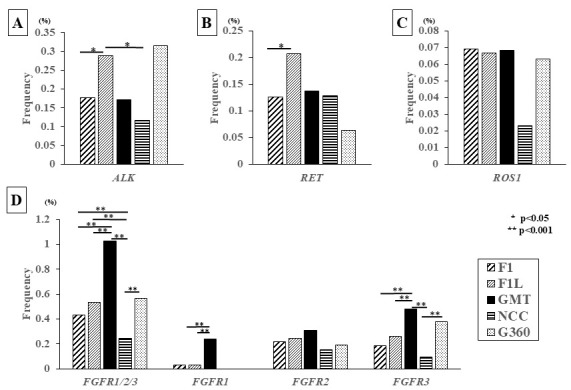
Fusion genes detected by multi-CGP test. (**A**) The *ALK* fusion gene was detected most frequently in G360, followed by F1L, F1, GMT, and NCC, with a significant difference between F1L and F1/NCC (*p* < 0.05). The detection rate was higher in multi-CGP tests using blood samples than in those using tissue samples. (**B**) *RET* gene fusion was most frequently detected in F1L, with a significantly higher detection rate in F1L compared to F1 (*p* < 0.05). (**C**) There was no difference in the detection rate of *ROS1* gene fusion among the four multi-CGP tests except for NCC, and no significant difference was observed. (**D**) When comparing the detection rate of all *FGFR* fusion genes among multi-CGP tests, the detection rate by GMT was significantly higher than that of F1/F1L/NCC (*p* < 0.001). Compared by gene, the detection rate for *FGFR1* and *FGFR3* was highest with GMT, and significant differences were also observed among the other multi-CGP tests. No significant difference was observed for the *FGFR2* fusion gene, and the detection rate also showed little difference among the multi-CGP tests. * *p* < 0.05; ** *p* < 0.001.

Figure 4 shows the detection frequency of gene fusions in four genes other than *NTRK* that are associated with therapeutic drugs available under insurance in Japan (*ALK*, *RET*, *ROS1*, *FGFR1/2/3/4*). A total of 169 cases were found to show *ALK* fusion genes (Supplementary Materials, Figure S1). *EML4* was a frequent fusion partner (63.9%, 108/169). The organ in which *ALK* fusion was most frequently detected was the lung (52.0%, 88/169 cases), followed by the bowel (14.7%, 25/169 cases). A total of 57 cases were found to show *ROS1* fusion genes (Supplementary Materials, Figure S2). Both *CD74* and *GOPC* were frequent fusion partners (19.2%, 11/57 cases), respectively. The organ in which *ROS1* fusion was most frequently detected was the lung (38.5%, 22/57 cases). A total of 124 cases were found to show *RET* fusion genes (Supplementary Materials, Figure S3). *KIF5B* was a frequent fusion partner (27.4%, 34/124 cases), followed by *CCDC6* (25.8%, 32/124 cases). The organ in which *RET* fusion was most frequently detected was the lung (45.9%, 57/124 cases), followed by the thyroid (20.1%, 25/124 cases). The detection rates of ALK fusion genes were 0.18% (110 of 62,105 cases) for F1, 0.29% (39 of 13,475 cases) for F1L, 0.17% (5 of 2926 cases) for GMT, 0.12% (10 of 8597 cases) for NCC, and 0.32% (5 of 1585 cases) for G360. The multi-CGP test that most frequently detected ALK fusion genes was G360, but there were no significant differences between the other multi-CGP tests. However, the detection rate by F1L, which had the second highest detection frequency, was significantly higher than that of F1/NCC (Figure 4A). Next, the detection rates of RET fusion genes were 0.13% (78 of 62,105 cases) for F1, 0.21% (28 of 13,475 cases) for F1L, 0.14% (4 of 2926 cases) for GMT, 0.13% (11 of 8597 cases) for NCC, and 0.06% (1 of 1585 cases) for G360. Therefore, F1L was the most common detection method. When compared with other multi-CGP tests, a significant difference was only observed between F1L and F1, revealing that the gene was detected more frequently in blood than in tissue (Figure 4B). The detection rates of ROS1 fusion genes were 0.07% (43 of 62,105 cases) for F1, 0.07% (9 of 13,475 cases) for F1L, 0.07% (2 of 2926 cases) for GMT, 0.02% (2 of 8597 cases) for NCC, and 0.06% (1 of 1585 cases) for G360. There were little differences in the detection frequencies among the multi-CGP tests (Figure 4C). Finally, the detection rates of FGFR fusion genes are shown in Figure 4D. For FGFR fusion genes, the detection rate of GMT was the highest for FGFR1, FGFR2, and FGFR3, and the detection rate for FGFR1 and FGFR3 was significantly higher than that of other multi-CGP tests. Among five multi-CGP tests, the detection rates of ALK and RET fusion genes were significantly higher in F1L, and the detection rates of NTRK and FGFR fusion genes were significantly higher in GMT.

## 4. Discussion

The frequency of *NTRK1/2/3* gene fusions in solid tumors was 0.20% in all 88,688 patients of C-CAT data, which is similar to the frequency reported not only in Japan [19] but also in other countries [20,21]. *NTRK1/2/3* gene fusions were significantly more frequent in patients under 20 years of age (1.74%) compared to those over 20 years (0.17%), which indicated that the occurrence of *NTRK* gene fusions is involved in the development of young tumors. The primary sites of young tumors with *NTRK* gene fusions were frequently in soft tissue, the CNS/brain, kidneys, and bone, and the frequent histologic types were sarcoma and neuroblastoma. Since *NTRK* genes are frequently localized in the CNS, PNS, and smooth muscle, mutations might be more prevalent in younger people who are still in the differentiation stage, which might be one of the reasons why the prevalence was higher in these age groups than in adults.

Of the 175 cases, 90 cases were administered Trk/NTRK inhibitors. Disease control, defined as complete response (CR), partial response (PR), or stable disease (SD), was achieved in 52% (47/90) of *NTRK* fusion gene cases, with no differences between *NTRK1*, NTRK2, and *NTRK3*. Of these, 25 cases (61.0%) of *NTRK1*, 5 cases (41.7%) of *NTRK2*, and 17 cases (45.9%) of *NTRK3* achieved disease control. Among the cases administered Trk/NTRK inhibitors, 16 cases in which *NTRK* gene fusion was detected in metastatic lesions also achieved disease control. These findings suggested that Trk/NTRK inhibitors showed useful therapeutic efficacy for patients with *NTRK* fusion genes.

*NTRK* gene fusions were frequently detected in the head and neck, soft tissue, thyroid, intestine, and brain. All of the head and neck tumors with *NTRK* gene fusion showed *ETV6*-*NTRK3*, which has been reported to be frequently detected in secretory tumors, including salivary gland cancer [22,23,24]. The most common histological type of head and neck tumors was mammary analog secretory carcinoma of salivary gland origin, suggesting that *ETV6*-*NTRK3* might be associated with secretory tumors. Trk/*NTRK* inhibitors might be promising for head and neck tumors, especially secretory tumors such as salivary gland cancer.

*NTRK* gene fusions were more frequently detected by the use of tissue samples compared to blood samples. *NTRK* gene fusions were detected in metastatic tumors among those treated with Trk/NTRK inhibitors who achieved disease control. More than half of the tissue samples submitted had been collected within the past year, but the fusion genes were also detected in tissue samples that were more than five years old. These findings might suggest that submitting tissue samples to the multi-CGP tests was useful for detecting *NTRK* gene fusions not only in primary tumors but also in metastatic tumors. In contrast, since Trk/NTRK inhibitors are promising for the patients with *NTRK* gene fusions, the liquid multi-CGP test, such as F1L by blood samples, might be useful when tissue collection is difficult.

The detection rate of *NTRK* gene fusions was significantly higher with an RNA assay than with a DNA assay, and GMT was found to be the most useful test of the current five multi-CGP tests. Among *NTRK* gene fusions, *NTRK2* and *NTRK3* are considered to be more difficult to identify than *NTRK1* in terms of fusion breakpoints; *NTRK2* and *NTRK3* contain larger genomic regions compared to *NTRK1*, and it has been reported that detection rates are particularly low when searching using DNA-targeted analysis methods [25]. It is technically difficult to design and map these large intron regions for optimal DNA hybridization capture by DNA assay, and this resulted in low sensitivity for the DNA fusion point. In contrast, RNA assay showed high sensitivity for fusion genes because the exon junctions of the fusion gene transcripts are directly detectable [26,27]. These findings might suggest that GMT with RNA assay is effective for the detection of *NTRK* fusions, especially for *NTRK2/3* gene fusions.

Among the multi-CGP tests using a DNA assay, F1/F1L only detected all exons and introns in 12 regions of *NTRK2* and all exons in *NTRK3*, which resulted in the partial detection of the intron regions that were abundant in *NTRK2/3*. On the other hand, NCC, which had a relatively high detection rate among DNA assays, was used to analyze the entire *NTRK2/3* region and showed a higher detection rate of *NTRK2/3* fusions because more intron regions were searched for using NCC in comparison to F1/F1L. The detection of *NTRK3* fusions by DNA assay is considered to be difficult because of the many fusion partners that arise in *NTRK3* fusion cases. On the other hand, because *NTRK2* has a complex structure and the detection rate of *NTRK2* fusion is low, the implementation of multi-CGP tests using RNA assays, including GMT, may lead to the elucidation of the *NTRK2* fusion mechanism in the future.

When comparing the detection rates of other fusion genes such as *ALK*, *RET*, *ROS*, and *FGFRs* by each multi-CGP test (Supplementary Materials, Figures S1–S3), a different detection rate of *FGFR1* fusion and *FGFR3* fusion was found between RNA assay and DNA assay. Because no specific breakpoint detection has been found in *FGFR1* and *FGFR3*, the detection rate for the fusion of *FGFR1* and *FGFR3* may be significantly higher in an RNA assay such as GMT. In contrast, Pemigatinib or Futibatinib, which target *FGFR2* fusion genes, have been recently approved in Japan. Since *FGFR2* has had a lot of breakpoints and fusions [28], many more designs of FGFR2 breakpoints might be necessary for multi-CGP tests in order for them to be frequently detectable by DNA assay, which may result in no difference in the detection rates of *FGFR2* fusion between the RNA assay and the DNA assay in the near future.

On the other hand, the detection rate of *ALK* gene fusions and *RET* gene fusions was significantly higher in F1L. *ALK* gene fusions have been reported frequently in lung cancer, especially in non-small-cell lung cancer, and *EML4*-*ALK* is known to be the most frequent driver gene [29]. Also, it has been reported that the detection of various breakpoints and fusion partners of *ALK* gene fusions from blood samples in lung cancer patients is clinically useful [30,31]. In this study, the organs in which *ALK* gene fusion was detected were the lung, followed by the intestine, with *EML4*-*ALK* accounting for 78.7% of the total. The F1L test for *ALK* analysis was set to the entire exon and intron 18/19 region, including the *EML4*-*ALK* breakpoint. Furthermore, the detected lung and intestine are prone to hematogenous metastasis, and tumor components are likely to appear in the blood, suggesting that the detection rate was high in liquid multi-CGP tests using blood samples. The high detection rate in blood samples might be due to the high frequency of hematogenous metastasis. RET gene fusions have been reported as driver genes for various types of cancer, including thyroid, lung, intestine, pancreas, and breast, and fusions such as *CCDC6*-*RET*, *NCOA4*-*RET*, and *KIF5B*-*RET* are known to be frequently seen [32,33]. It has been reported that breakpoints of RET fusion exist in the intron 7–11 [28]. F1/F1L might detect *RET* fusions sufficiently because F1/F1L is designed to detect RET fusion at the intron 7–11 regions.

The breakpoints and fusion partners are important for determining the various gene fusions. Because the detection rate in DNA assays depends on the complexity of the gene structure, information on breakpoints and fusion partners is necessary for multi-CGP tests using DNA assays. On the other hand, RNA assays show high sensitivity for fusion genes because the exon junctions of the fusion gene transcripts are relatively detectable.

## 5. Conclusions

In conclusion, among the currently available multi-CGP tests, GMT with RNA sequencing analysis might show a favorite diagnostic ability for gene fusions, especially for *NTRK* fusion.

## Figures and Tables

**Table 1:** Correlation between *NTRK1/2/3* fusion and clinical characteristics of patients in 88,688 tumors.

Clinical Features	*NTRK1/2/3* Fusion			*NTRK1* Fusion			*NTRK2* Fusion			*NTRK3* Fusion		
	Positive	Negative		Positive	Negative		Positive	Negative		Positive	Negative	
	*n* = 175	*n* = 88,513	*p*-Value	*n* = 83	*n* = 88,605	*p*-Value	*n* = 21	*n* = 88,667	*p*-Value	*n* = 71	*n* = 88,617	*p*-Value
Sex												
Male (*n* = 44,337)	95 (0.21%)	44,242 (99.79%)		36 (0.08%)	44,301 (99.92%)		14 (0.03%)	44,323 (99.97%)		45 (0.10%)	44,292 (99.90%)	
Female (*n* = 44,346)	80 (0.18%)	44,266 (99.82%)	n.s.	47 (0.11%)	44,299 (99.89%)	n.s.	7 (0.02%)	44,339 (99.98%)	n.s.	26 (0.06%)	44,320 (99.94%)	0.02
Age												
<20 (*n* = 1670)	29 (1.74%)	1641 (98.26%)		14 (0.84%)	1656 (99.16%)		3 (0.18%)	1667 (99.82%)		12 (0.72%)	1658 (99.28%)	
≥20 (*n* = 87,018)	146 (0.17%)	86,872 (99.83%)	<0.001	69 (0.08%)	86,949 (99.92%)	<0.001	18 (0.02%)	87,000 (99.98%)	<0.001	59 (0.07%)	86,959 (99.93%)	<0.001
Multi-CGP tests assay												
DNA assay (*n* = 85,762)	155 (0.18%)	85,607 (99.82%)		78 (0.09%)	85,684 (99.91%)		14 (0.02%)	85,748 (99.98%)		63 (0.07%)	85,699 (99.93%)	
RNA assay (*n* = 2926)	20 (0.68%)	2906 (99.32%)	<0.001	5 (0.17%)	2921 (99.83%)	n.s.	7 (0.24%)	2919 (99.76%)	<0.001	8 (0.27%)	2918 (99.73%)	<0.001
Materials												
Tissue (*n* = 73,628)	157 (0.21%)	73,471(99.79%)		71 (0.10%)	73,557 (99.90%)		19 (0.03%)	73,609 (99.97%)		67 (0.09%)	73,561 (99.91%)	
Blood (*n* = 15,060)	18 (0.12%)	15,042 (99.88%)	0.02	12 (0.08%)	15,048 (99.92%)	n.s.	2 (0.01%)	15,058 (99.99%)	n.s.	4 (0.03%)	15,056 (99.97%)	0.01

## Data Availability

The datasets presented in this article are not readily available because the data are part of an ongoing study or due to limitations. Requests to access the datasets should be directed to the Center for Cancer Genomics and Advanced Therapeutics (C-CAT).

## Supplementary Materials

The following supporting information can be downloaded at: https://www.mdpi.com/article/10.3390/cancers17132250/s1, Figure S1: Fusion partners for ALK; Figure S2: Fusion partners for ROS1; Figure S3: Fusion partners for RET; Table S1: The number of primary tumors with NTRK fusion in each multi-CGP test according to age.
