# Supplementary material for: Multi-Cancer Genome Profiling for Neurotrophic Tropomyosin Receptor Kinase (NTRK) Fusion Genes: Analysis of Profiling Database of 88,688 Tumors
Source: Cancers (Basel). 2025 Jul 4;17(13):2250. doi: 10.3390/cancers17132250 (PMC12248648; doi:10.3390/cancers17132250)
Supplement: Supplementary file 1 [file cancers-17-02250-s001.zip › cancers-3699344-supplementary.pdf]

Supplement Figure S1. Fusion partners for ALK.

Fusion partner gene / tumor type	*ALK*																																																	
	Total	*ADAMTS2*	*AFF3*	*ALMS1*	*ATIC*	*BRE*	*CEP44*	*CGREF1*	*CIB4*	*CTSB*	*DCTN1*	*DTNB*	*EGFR*	*EML4*	*EML6*	*EXOC6B*	*FN1*	*FOPNL*	*GALNT14*	*GPHN*	*HIP1*	*HMBOX1*	*HOOK1*	*IAH1*	*IGFBP5*	*KIAA1217*	*KIF5B*	*MYH9*	*NF1*	*PLEKHH2*	*PPP1R21*	*PPP2R3A*	*RANBP2*	*RBBP8*	*ROCK2*	*RRBP1*	*SEMA5A*	*SLC12A2*	*SLC5A6*	*SNTG2*	*SPTBN1*	*STRN*	*THUMPD2*	*TNS1*	*TPM3*	*TRIM13*	*TRIM24*	*TRIM25*	*USO1*	*USP34*
**Total**	**169**	**1**	**1**	**1**	**1**	**1**	**1**	**1**	**1**	**1**	**1**	**1**	**1**	**108**	**1**	**1**	**1**	**1**	**1**	**1**	**2**	**2**	**1**	**1**	**2**	**1**	**4**	**2**	**1**	**2**	**1**	**1**	**1**	**1**	**1**	**1**	**1**	**1**	**1**	**1**	**2**	**5**	**1**	**1**	**1**	**1**	**1**	**1**	**1**	**1**
**CNS/Brain**	7				1						1			2		1			1																1															
**Thyroid**	4									1				1												1					1																			
**Lung**	88					1								79							2			1			2			1						1							1							
**Esophagus/Stomach**	3											1		2																																				
**Ampulla of Vater**	2																					1						1																						
**Bowel**	25	1		1			1		1				1	10	1			1		1														1				1			2	1				1			1	
**Biliary Tract**	9													3															1													3			1					1
**Kidney**	1													1																																				
**Bladder/Urinary Tract**	1																1																																	
**Pancreas**	4													3									1																											
**Skin**	2													1																							1													
**Ovary/Fallopian Tube**	1													1																																				
**Uterus**	3																								1							1												1						
**Prostate**	4		1					1														1																	1											
**Soft Tissue**	9													2											1		1	1		1			1							1							1			
**Peritoneum**	1																																									1								
**Other**	5													3													1																					1		

Supplement Figure S1. A total of 169 cases was shown *ALK* fusion genes. *EML4* was a frequent fusion partner (63.9％, 108/169). The organ in which *ALK* fusion was most frequently detected was the lung (52.0%, 88/169 cases), followed by Bowel (14.7%, 25/169 cases).

Supplement Figure S2. Fusion partners for ROS1.

	ROS1																													
**tumor type \ Fusion partner gene**	**Total**	***AFAP1L2***	***ANKRD6***	***CCDC30***	***CD74***	***CEP85L***	***CNNM3***	***DSE***	***EEF1G***	***EGFR***	***FRK***	***GOPC***	***HBS1L***	***IGH***	***KDELR2***	***KIAA1244***	***MAP3K9***	***MYO5C***	***PEX3***	***RAB3IP***	***SBF2***	***SDC4***	***SLC34A2***	***SLC35F1***	***SLC4A4***	***SLMAP***	***TNS1***	***TPM3***	***USP45***	***VGLL2***
**Total**	**57**	**1**	**1**	**1**	**11**	**2**	**1**	**1**	**1**	**1**	**1**	**11**	**1**	**1**	**1**	**1**	**1**	**1**	**1**	**1**	**1**	**5**	**2**	**1**	**3**	**1**	**1**	**1**	**1**	**1**
**CNS/Brain**	**3**	**1**				**1**			**1**																					
**Thyroid**	**1**			**1**																										
**Lung**	**22**				**11**						**1**				**1**			**1**				**5**	**2**					**1**		
**Esophagus/Stomach**	**4**					**1**				**1**		**2**																		
**Ampulla of Vater**	**1**											**1**																		
**Bowel**	**6**											**3**					**1**		**1**					**1**						
**Biliary Tract**	**2**							**1**																		**1**				
**Pancreas**	**6**						**1**																		**3**				**1**	**1**
**Ovary/Fallopian Tube**	**2**											**2**																		
**Breast**	**2**		**1**									**1**																		
**Uterus**	**2**											**1**															**1**			
**Prostate**	**1**											**1**																		
**Soft Tissue**	**3**												**1**			**1**				**1**										
**Peritoneum**	**1**													**1**																
**Other**	**1**																				**1**									

Supplement Figure S2. A total of 57 cases was shown *ROS1* fusion genes. Both *CD74* and *GOPC* were frequent fusion partner (19.2%, 11/57 cases), respectively. The organ in which *ROS1* fusion was most frequently detected was the lung (38.5%, 22/57 cases).

Supplement Figure S3. Fusion partners for RET.

RET																																					
**Fusion partner gene / tumor type**	**Total**	***ACPP***	***ANKRD26***	***ARHGAP12***	***C10orf53***	***CCDC6***	***CGNL1***	***ERC1***	***ETV6***	***FAP***	***GOLGB1***	***HNRNPA3P1***	***KIAA1199***	***KIAA1217***	***KIAA1468***	***KIF5B***	***KTN1***	***LINC00839***	***MOBP***	***MYH10***	***MYH9***	***NCOA4***	***NEBL***	***NRG3***	***PCM1***	***PCNXL2***	***PDE5A***	***PRKAR1A***	***PRKG1***	***PWWP2A***	***RASGEF1A***	***RRBP1***	***SSBP2***	***TRIM27***	***TRIM33***	***VCAN***	***ZMIZ1***
**Total**	**124**	**1**	**1**	**1**	**1**	**32**	**1**	**2**	**1**	**1**	**1**	**1**	**1**	**2**	**2**	**34**	**1**	**1**	**1**	**1**	**1**	**19**	**1**	**1**	**2**	**1**	**1**	**1**	**2**	**1**	**2**	**1**	**1**	**1**	**1**	**1**	**1**
**CNS/Brain**	**2**																																			**1**	**1**
**Head and Neck**	**2**								**1**													**1**															
**Thyroid**	**25**					**12**					**1**				**1**		**1**					**7**					**1**				**1**		**1**				
**Lung**	**57**		**1**			**12**	**1**	**1**		**1**				**1**		**32**			**1**			**2**	**1**	**1**				**1**	**1**						**1**		
**Esophagus/Stomach**	**3**					**2**																				**1**											
**Bowel**	**15**				**1**	**3**							**1**								**1**	**7**			**1**							**1**					
**Biliary Tract**	**3**					**1**		**1**														**1**															
**Liver**	**1**													**1**																							
**Pancreas**	**4**														**1**							**1**			**1**					**1**							
**Skin**	**1**															**1**																					
**Ovary/Fallopian Tube**	**1**											**1**																									
**Breast**	**3**					**1**										**1**		**1**																			
**Prostate**	**3**	**1**																											**1**		**1**						
**Soft Tissue**	**1**																			**1**																	
**Peritoneum**	**1**					**1**																															
**Other**	**2**			**1**																														**1**			

Supplement Figure S3. A total of 124 cases was shown *RET* fusion genes. *KIF5B* was a frequent fusion partner (27.4%, 34/124 cases), followed by *CCDC6* (25.8%, 32/124 cases). The organ in which *RET* fusion was most frequently detected was the lung (45.9%, 57/124 cases), followed by Thyroid (20.1%, 25/124 cases).

Supplement Table S1.

		patients of <20 years old					patients of 20≥years old				
		F1	F1L	CC	G360	GMT	F1	F1L	NC	G360	GMT
Adrenal Gland	Adrenocortical Carcinoma								1		
Biliary Tract	Biliary Tract						1				
	Gallbladder Adenocarcinoma, NOS						1				
	Intrahepatic Cholangiocarcinoma							2			
	Perihilar Cholangiocarcinoma						1				
Bone	Chondroblastic Osteosarcoma	1									
	Dedifferentiated Chordoma										1
	Osteosarcoma	1									
Bowel	Colon Adenocarcinoma						5	1			
	Colorectal Adenocarcinoma						1				
	Duodenal Adenocarcinoma						1				
	Mucinous Adenocarcinoma of the Colon and Rectum						2				
	Rectal Adenocarcinoma							2	1		1
Breast	Breast Invasive Ductal Carcinoma						3	1			
	Invasive Breast Carcinoma							1			
	Juvenile Secretory Carcinoma of the Breast						2				
Cervix	Cervical Adenosquamous Carcinoma										1
CNS/Brain	CNS/Brain										1
	Diffuse gliomas					1					1
	Glioblastoma Multiforme										1
	High-Grade Glioma, NOS	1									
	Pilocytic Astrocytoma	2				1					
Esophagus/Stomach	Adenocarcinoma of the Gastroesophageal Junction						1				
	Esophageal Squamous Cell Carcinoma						2				
	Intestinal Type Stomach Adenocarcinoma						1				
	Tubular Stomach Adenocarcinoma						1				
Head and Neck	Acinic Cell Carcinoma						3				
	Head and Neck						2				
	Mammary Analogue Secretory Carcinoma of Salivary Gland	1					1		1		2
	Origin						1				
	Myoepithelial Carcinoma							1			
	Salivary Adenocarcinoma						1				
	Salivary Carcinoma						1				1
	Salivary Carcinoma, Other						1				
	Salivary Duct Carcinoma						1				
	Sinonasal Adenocarcinoma								1		
Kidney	Kidney	1									
	Unclassified Renal Cell Carcinoma						1				
	Wilms' Tumor					1					
Liver	Hepatocellular Carcinoma								1		
Lung	Lung								1		
	Lung Adenocarcinoma						3	1	1		1
	Lung Squamous Cell Carcinoma						1				
Other	Adenocarcinoma, NOS						1				
	Cancer of Unknown Primary						1				
	Other								1		
	Undifferentiated Malignant Neoplasm			1							
Ovary/Fallopian Tube	High-Grade Serous Ovarian Cancer						1				
	Ovarian Cancer, Other						1				
Pancreas	Pancreas							1			
	Pancreatic Adenocarcinoma						3		2		
	Pancreatic Neuroendocrine Carcinoma						1				
Peripheral Nervous System	Malignant Peripheral Nerve Sheath Tumor						1				1
Prostate	Prostate Adenocarcinoma						3	5	1		
Skin	Skin								1		
	Spitzoid Melanoma	1									
	Sweat Gland Carcinoma/Apocrine Eccrine Carcinoma						2				
Soft Tissue	Angiomatoid Fibrous Histiocytoma						1				
	Angiosarcoma						1				
	Dedifferentiated Liposarcoma						1				
	Ewing Sarcoma of Soft Tissue						1				
	Gastrointestinal Stromal Tumor						1	1			
	Infantile Fibrosarcoma	3		1		1					
	Leiomyosarcoma						1				
	Round Cell Sarcoma, NOS					1					
	Sarcoma, NOS	2	1			1	1		1		
	Soft Tissue	1					2				
	Well-Differentiated Liposarcoma						1				
Testis	Testis						1				
Thyroid	Follicular Thyroid Cancer							1			
	Papillary Thyroid Cancer					1	9		6		2
	Thyroid						1				
Uterus	Uterine Epithelioid Leiomyosarcoma						1				
	Uterine Serous Carcinoma/Uterine Papillary Serous Carcinoma						1				
